# Dr. Reid, on Cold Ablution in Scarlatina

**Published:** 1804-01-01

**Authors:** J. Reid

**Affiliations:** Southampton Row


### *To the Editors of the Medical and Physical Journal.*

GENTLEMEN,

THE following Cases of Scarlatina, in which cold and tepid ablution were tried with unequivocal efficacy and success, occurred in the practice of the Finsbury Dispensary, under the care of myself and that of my friend and then assistant, Dr. Murray, now of Knaresborough. — I have been prevented by a propensity to procrastination, from before offering them to your acceptance, of which, it is hoped, they may not be deemed altogether unworthy.

*Southampton Row,*

*Nov.* 21, 1803.

I am, &c.
J. REID.

---

Charles Haywood, ætat. eight years, on May 24, 1803, was attacked with severe pain of head, accompanied by shiverings and general uneasiness. On the following day he was visited by us; his head-ach and febrile anxiety had greatly augmented; he complained of soreness in his throat; and upon inspection, the tonsils were found to be much inflamed, and of a deep red colour. His face, breast, and legs had assumed a bright red colour, from an eruption which had broken out that morning. He had lost his appetite. Tongue foul, but moist; eyes dull and heavy; body open; pulse quick, small, but regular; spirits little affected; skin hot and dry. He was ordered to take one table spoonful of a solution of two grains of tartar emetic in four ounces of water, and this to be repeated every half hour until full vomiting should take place; and it was directed that his whole body should be washed with tepid water, and afterwards carefully dried. This was repeated thrice a day.

28. The affection of throat continuing very severe, a blister was yesterday applied to the nape of his neck, and he frequently gargled with sage tea and vitriolic acid. He was washed as directed, and expressed much satisfaction after it, feeling himself less uneasy than he previously had been; in particular, after the first and third washings he fell into a fine calm sleep. Pulse less frequent and of better strength; throat still painful, but not so much inflamed; skin cool and relaxed; bad head-ach; eruption scaling off; appetite began to improve, and he was ordered to take porter.

30th.

30th. Washing had been continued till this day, when it was left off, a further perseverance in it being deemed unnecessary, the febrile symptoms having left him. Throat almost well, redness of skin having disappeared; a desquamation of the cuticle, as usually happens, succeeded; appetite good, skin cool, pulse 80, and regular; body regular. Being very feeble, the mist. Peruv. acidul. of the Dispensary was ordered to the extent of a table spoonful three times in the course of the day. This mixture consists of half an ounce of the bark and half a drachm of sulphuric acid to half a pound of water.

Jane Haywood, æt. 22, on the 30th of May was seized with pain of head, nausea, shivering, and soreness of throat. These complaints became worse on the following day, and a bright red eruption came out on her body. She took gr. 15 of ipecacuanha, which operated fully, and had a blister to the nape of her neck.

June 3. Throat much inflamed, but not swelled; pulse 100, small and quick; skin hot, dry, and tense; eruption perfectly diffused, without any distinct spots; she appeared to be extremely feeble and low; tongue very foul, but moist; body regular. She took two table spoons full of the solution of tartar emetic, and vomited gently. She was washed with tepid water, in the same manner as was directed for the first mentioned patient, who is her brother, and felt very comfortable (as she expressed it) after each sponging. She had few bed clothes on her, and her chamber was kept well aired. She used the same gargle as her brother did, but would hardly take any nourishment, and in particular, strenuously refused wine and porter.

5. The washing was still continued three times in the day; and from being uneasy and very hot, she became cool and less irritable after the sponging; the inflammation of her throat was also much relieved; pulse 80, regular, but small. Her appetite began to return this day, and she ate pudding and a boiled egg with great relish. She was directed to take two table spoons full of the mist. Peruv. and on the next day found herself so much better as to come to the Dispensary, a distance of half a mile from where she resided. Fortunately no bad consequence followed this act of imprudence, and she recovered strength with rapidity.

Mary Haywood, æt. 20, was attacked with all the usual symptoms on May 12, about a fortnight previous to the illness illness of her sister Jane. The eruption appeared on the fourth day of the disease, and had disappeared on the 18th of May, when we first saw her. She was then in a considerable stupor, had great dyspnœa, and seemed to have lost the power of swallowing from the passage being loaded with phlegm. Mouth and tongue extremely foul; lips black; pulse very frequent, but so feeble as not to be exactly numbered; skin burning hot, and very dry; body open; face pale, and shrunk; rattling of the mucous in her throat; the torpor was, at times, interrupted by violent fits of delirium. A large blister was immediately applied to the nape of the neck, which having begun to rise about three o'clock next morning, two table spoons full of the solution of tartar emetic were poured down her throat, and produced moderate vomiting. Her face, neck, and feet were likewise twice sponged with tepid water.

19. She began to recover perception, power of moving, and swallowing, soon after the blister had taken effect. Mouth and tongue much cleaner; pulse small, and 100, but much stronger and more regular than at the first visit; skin cooler. The washing was repeated; and a tendency to the torpor having recurred, her feet were immersed in warm water for nearly an hour.

James Haywood, ætat. 18, after the usual symptoms of fever and sore throat, had the eruption appear on his body on May 30, being the third day of his illness. He took an emetic of fifteen grains of ipecacuanha, and had a blister to the back part of his neck, which having risen extremely well, gave great relief to the affection of the throat. He was twice a day washed with tepid water.

June 3. Although this was the seventh day of the disease, and the eruption had disappeared, yet as his skin was very hot and arid, and his pulse remarkably full and strong, and very frequent, he was this day thrice washed with cold water. His throat being quite free from inflammation, no further remedies were thought necessary.

5. He was washed three times yesterday with cold water, and felt himself much relieved; his pain of head was removed; his skin became moderately relaxed and cool; pulse 80, and of good strength. He was dismissed convalescent.

Mary Norris, ætat. 9, was taken ill on July 29th, 1802. The day of the appearance of the scarlet eruption could not be learnt, but it was completely out on Aug. 2, (fifth day day of the fever). Her throat was much inflamed, and somewhat swelled; pulse quick and feeble; body open; skin hot and dry. She took two table spoons full of the solution of tartarised antimony, which operated to full vomiting; she had a blister to the nape of her neck, and gargled with sage tea and acid. She was washed with cold water, was kept perfectly cool, and allowed to remain but very little in bed during the day.

4. She also felt more comfortable after each washing. Her skin was relaxed, and of natural heat; pulse still quick, and about 80, but of much better strength. Washing, of course, was discontinued; nor did she require any other remedies.

Two of her brothers were also affected with scarlet fever, which run a course very similar to that of her's; and as they were treated precisely in the same manner, and washed with cold water, it was deemed to be superfluous to give a longer account of their cases.

John Blackwell, ætat. 15, was first seen on August 18, being the sixth day since the commencement of his disorder. His skin had assumed a dark red colour, from an eruption that had come out on the third day of the fever, and which, as usual, was entirely diffused, without any distinct, separate pimples. Throat much inflamed, and swelled; tongue white, and parched; skin hot; pulse very quick, and rather strong. He had an emetic given him early in the disease. He was directed to be washed with cold water, and had a blister applied to the nape of his neck. He gargled often with the common gargle.

20. Was washed eight times. The eruption had disappeared. Pulse 74, and of natural strength; appetite much improved; heat of skin, and general sense of uneasiness, removed; pain of head and soreness of throat gone; appetite beginning to improve; body open. No other medicines employed.

His sister, aged 18, also had scarlatina, and was visited on the second day of the fever. She was washed with cold water; but having a very trifling cynanche, no other remedies were administered, except an emetic. She was free from febrile symptoms on the third day.

Sarah Callad, ætat. 16, was admitted a patient on Sept. 17th, being the seventh day since her attack. Affection of throat very severe; considerable eruption remaining on her body; great febrile anxiety; nausea; sickness; pain of of head; skin hot and tense; pulse small, frequent, and irregular. She had a blister to her neck and gargled frequently, which afforded relief to her throat. She was washed repeatedly with tepid water, and the fever left her on the 19th; but as she continued very weak, she took the bark mixture.

Richard Webb, ætat. nineteen, on September 18, was attacked with shiverings, head-ach and slight nausea, followed on the next day by soreness of throat and difficulty in swallowing.

21. The eruption appeared mostly on his breast. He was first seen on the 22d, when his pulse was quick and small, skin hot, thirst very urgent, body regular, appetite lost; throat much swelled; and was treated in the usual way. He was washed with cold water; had nourishing diet and wine and water; he likewise took fifteen grains of ipecacuanha.

24. He was almost free from fever; scarlet eruption had disappeared. The convalescence of this patient was tedious, and he had a swelling of the sub maxillary glands which proved very troublesome.

John Oxden, aged four years, on October 13, was seized with pain of head, shivering, sickness, and soreness of throat, succeeded on the third day by a bright red eruption on his breast and belly.

October 15. Throat much inflamed and swelled, intense pain of head, skin very hot and dry, pulse quick and small, but regular; body open. He was ordered to be washed with cold water, and kept very cool, with few bed-clothes, and the window of his room constantly open. He took ten grains of ipecacuanha without effect; and to gargle as usual.

18. Owing to the very blameable prejudice of his mother he was not washed, although most particularly directed; he was likewise oppressed by the weight of his bed-clothes; and the window of the chamber in which he lay, was kept open only at intervals during the day. His pulse was extremely feeble and irregular; eruption as before; pain of head very severe; throat very painful, and much swelled; mouth and tongue exceedingly foul. He was delirious the preceding night, and refuses to take any medicine or drink. He frequently screams violently, and at other times lay in an imperfect torpor; two tea-spoons full of the solution of emetic tartar were poured down his throat; this was again repeated, when it produced slight vomiting; but he could not be made to swallow more either of this or diluted sulphuric acid. A blister was put to the nape of his neck, and he was once washed with tepid water; but all in vain, for he died early next morning. The eruption, after death, passed most rapidly into gangrene.

Frances Game, ætat. nine, was first admitted as a patient on November 15, being the eleventh day of the fever. Eruption very trifling, and disappearing at intervals; excessive pain of head, with considerable soreness of her throat and mouth; and upon inspection, the inflammation of the tonsils was perceived to have spread to the internal fauces. Tongue red and foul. The inflammatory affection was removed by the use of remedies similar to those employed in the before mentioned patients. Her body was washed with warm water.

17. The fever had abated; her pulse was soft and of good strength, heat natural; but the general anxieties and the head-ach still continued. She was very feeble and had a very bad appetite. The washing was continued, and she began to take the Peruvian bark.

19. She was free from fever; but the pain of head still being very troublesome, blisters were applied to her temples, with the best effect.

Continuing to take the bark, she improved in strength and appetite, although slowly.

John Crick, ætat. 10. In this patient the usual symptoms were succeeded by the eruption on the fourth day, which hardly remained visible for two days. He was visited on December 22, the sixth day of his disorder, and no traces of the eruption could then be discerned. He was extremely low in spirits, and had a dread of death truly extraordinary in so young a person. He was very feeble, and could scarcely be kept the an erect posture without fainting; his skin was somewhat hot and dry; pulse very small and 90; tonsils of the throat of a florid colour, and rather swelled; appetite bad. A blister was applied to his neck, and afforded him much relief; he took a spoonful of emetic tartar, which operated mildly. His body was washed with tepid water twice in the course of the day; he drank red wine.

23. He was free from fever, but was left in a weak condition, and the fear of death still kept possession of his mind; mind; skin cool and moist; pulse 88, and regular; he had very disturbed and uneasy sleep. He now took the bark and red wine and water freely. His convalescence was tedious, but was not attended with any dropsical swellings.

His sister, 13 years old, caught scarlatina, and was washed likewise with warm water. Her recovery was rapid.

John Vaughan, ætat. 14, was first admitted as a Dispensary patient on January 21, 1803, having been ill for seven or eight days. He had taken two emetics; and, on the sixth day of the fever, had a blister to the nape of his neck. When we first saw him his thighs and legs were of a bright red colour; his pulse was very quick, irregular, and so feeble as not to be accurately numbered, it was 130 at least; skin hot and tense; tonsils of a deep red colour, and had several sloughs; great prostration of strength, and sense of general pain; tongue more red than natural, and dry; no appetite; body open. He was exceedingly deformed in person, and diminutive in size. His body was washed with tepid water; he used a gargle composed of half an ounce of myrrh, thirty drops of muriatic acid, and half a pound of water. He was ordered to take red wine freely, and to have two table spoons full of the acidulated bark mixture twice a day.

24. Fever nearly gone; scarlet eruption still visible; throat much relieved; he found himself easier; glowed considerably after the washings.

26. He was free from febrile symptoms, but remained very weak; however, in a day or two, his appetite returned, and he regained strength with rapidity.

Richard Vaughan, ætat. 11, having caught the scarlet fever from his brother, was visited on Jan. 28, the second or third day of his complaint. The eruption was copious, and of a very bright colour; pulse very quick and small; skin burning hot; tongue white, and dry; affection of throat very mild; an excessive sense of general uneasiness; sleeps very badly from his extreme irritability. He immediately took two spoons full of the antimonial solution, which occasioned full vomiting. He was washed with cold water, and had a pill of opium at bed time.

29. He washed twice, and found considerable relief from it. Heat natural; febrile anxiety diminished; eruption lessened in extent; bad sleep. He was ordered to take porter, and the washing was repeated.

31. The fever having completely left him, and his sleep and appetite having returned, he was considered as not requiring any further medical aid.

### Observations.

1. A peculiar recommendation of the above mode of treatment is, the rare occurrence of dropsical affections after the disease when thus managed, which in persons treated in the old method, so frequently supervene, and prove so very troublesome, and often dangerous.

2. In people of a scrophulous habit, swellings of the glands very often follow scarlet fever; but where the washing has been seasonably and vigorously employed, these seldom take place, even in persons having the *marks* which denote a scrophulous taint. In general, where the eruption is extensive, the sore throat is trifling, and vice versa.

3. The case of John Vaughan admirably illustrates the utility of washing, since two emetics and blisters had been previously tried with no benefit; but the disease yielded considerably after the two first washings, and was removed after this plan had been persevered in for two days longer.

4. It ought to be kept in mind, that in an early stage of the disease, when the strength is not as yet much reduced, when the skin is hot and dry, and where the febrile anxiety is considerable, *cold* washing is decidedly indicated. But, when extreme debility has come on, after the fever has continued for several days, when the pulse is small and irregular, the skin is more relaxed, then the re-action produced by cold washing might prove too violent, and, of course, in such cases, *tepid* sponging is preferable.

